# MicroRNA-338-3p suppresses tumor growth of esophageal squamous cell carcinoma *in vitro* and *in vivo*

**DOI:** 10.3892/mmr.2021.12450

**Published:** 2021-09-20

**Authors:** Xinyu Li, Zhihong Li, Guiyun Yang, Zhenxiang Pan

Mol Med Rep 12: 3951-3957, 2015; DOI: 10.3892/mmr.2015.3820

Following the publication of the above article, the authors have requested that it be retracted. After having repeated some of the experiments, the authors were not able to reproduce certain of the results. Furthermore, following a further investigation in the Editorial Office, it came to light that some of the wstern blotting data shown in Fig. 3 and the tumor images in Fig. 5 were strikingly similar to those that had been submitted for publication prior to the receipt of present article. Therefore, this article has been retracted from the Journal; all the authors agree to this retraction. The Editor and the authors would like to apologize for any inconvenience caused.

